# The utility of cemented femoral stems in modern THA: a 10-year comparative analysis of the Charnley and Exeter stems

**DOI:** 10.1007/s11845-023-03381-y

**Published:** 2023-05-27

**Authors:** Ailbhe White-Gibson, Gerard Anthony Sheridan, Adrian Cassar Ghetti, Peter Keogh, Paddy Kenny, James Patrick Cashman

**Affiliations:** The National Orthopaedic Hospital, Cappagh, Dublin, Ireland

**Keywords:** Arthroplasty, Cemented prosthesis, Charnley prosthesis, Exeter prosthesis, Total hip replacement, 10-year outcomes

## Abstract

**Background:**

Total hip replacement (THR) is one of the most common surgical procedures performed worldwide. The controversy surrounding the relative merits of a cemented composite beam or cemented taper-slip stem in total hip replacement continues. Our aims primarily were to assess the 10-year outcomes of cemented stems using Charnley and Exeter prostheses with regional registry data and secondarily to assess the main predictors of revision.

**Methods:**

We prospectively collected registry data for procedures performed between January 2005 and June 2008. Only cemented Charnley and Exeter stems were included. Patients were prospectively reviewed at 6 months, 2, 5 and 10 years. The primary outcome measure was a 10-year all-cause revision. Secondary outcomes included ‘re-revision’, ‘mortality’ and functional ‘Western Ontario and McMaster Universities Osteoarthritis Index’ (WOMAC) scores.

**Results:**

We recorded a total of 1351 cases in the cohort, 395 Exeter and 956 Charnley stems. The overall all-cause revision rate at 10 years was 1.6%. The revision rate for Charnley stem was 1.4% and 2.3% revision rate for all Exeter stems with no significant difference noted between the two cohorts (*p* = 0.24). The overall time to revision was 38.3 months. WOMAC scores at 10 years were found to be insignificantly higher for Charnley stems (mean 23.8, *σ* = 20.11) compared to Exeter stems (mean 19.78, *σ* = 20.72) (*p* = 0.1).

**Conclusion:**

There is no significant difference between cemented Charnley and Exeter stems; they both perform well above the international average. The decline in the use of cemented THA is not fully supported by this regional registry data.

## Introduction

Total hip arthroplasty (THA) is one of the most comment and successful surgical procedures performed worldwide. The total number of hip replacements recorded in the UK National Joint Registry (NJR) continues to increase totalling just under 1 million replacements since data was first collected in 2003 [1]. The first Irish National joint registry report outlined that there were 3723 hip arthroplasty cases performed from 2014 to 2019 with 379 of those revision cases. The rates of revision were reported as 1.1% at 1 year due to infection in 28% of cases and periprosthetic fracture also in 28% of cases. Cemented hip arthroplasty stems were used in 40% of cases during this time frame [2].

Cemented arthroplasty stems can be split into composite beam design and those that function with a taper slip mechanism. Composite beam stems achieve stability by interlocking at all interfaces, achieving fixation between the stem and cement. Taper-slip stems achieve stability via controlled subsidence within the cement mantle [3]. Radiostereometic analysis has shown that polished double-tapered femoral implants, such as Exeter stems, subside within cement, with no movement occurring at the cement–bone interface. [4] Despite in vitro studies demonstrating the differences in stem fixation, most in vivo reports have failed to demonstrate any significant difference in outcome or survivorship between composite beam and taper-slip designs [5, 6].

The discussion surrounding the relative merits of cemented and cementless fixation for THA continues. There is evidence to suggest that uncemented fixation methods may lead to increasing rates of periprosthetic fracture [7, 8]. Supporters of cemented fixation will note the suitability of cemented implants for all age groups and all femur types, including capacious femoral canals, regardless of local anatomy [9].

Sir John Charnley pioneered the concept of low-friction arthroplasty with his fully cemented THA design which he implanted using high molecular weight polyethylene in 1962 [10, 11]. The Charnley THA (DePuy), based on the composite beam design concept, has been considered by many to be the gold standard against which all other devices are compared [12]. There has been a trend in recent years towards uncemented stem prostheses. In 2012, in the USA, 93% of all THAs were performed using cementless stem implants [13], 70% of stem implants were cementless in Norway in 2017 [14] and 90% in Italy in the same year [15]. It has also been demonstrated in the Swedish registry that the proportion of all cemented implants has dropped from 92 to 68% from 1999 to 2012 [16].

The Charnley hip replacement demonstrated reproducible results with high survival rates due to its low friction properties [17, 18]. It emerged as a reliable solution for pain relief, and its design remains relevant decades later with a 20-year survivorship of over 80% [19–21]. The original Exeter stem was first implanted in 1970 and had extremely positive long-term results [22]. The Exeter V40 stem was introduced in 2000, and long-term follow-up has demonstrated comparatively excellent results [23] functioning as a taper slip device within the PMMA mantle [24, 25].

The primary aim of our study is to assess the 10-year outcomes of cemented stems comparing Charnley and Exeter prostheses with prospectively collected regional registry data. The secondary aim was to assess the main predictors of revision with these two common cemented femoral stems.

## Methods

This was a retrospective cohort study with prospectively collected data from our institutional arthroplasty register. This electronic institutional registry was established in February 2005 and has been maintained prospectively. Each patient undergoing primary THA with a minimum of 10-year follow-up data between January 2005 and June 2008 was eligible for inclusion. Post-operatively clinical review was performed at 6 months, 2 years, 5 years, and 10 years. Clinical and radiological assessments were performed at each follow-up and recorded. Exclusion criteria included cases with incomplete data collection as well as metal-on-metal implant types.

The primary outcome measure was a 10-year all-cause revision. Secondary outcomes included ‘re-revision’, ‘mortality’ and functional ‘Western Ontario and McMaster Universities Osteoarthritis Index’ (WOMAC) scores. We obtained ethical approval from the ethics at National Orthopaedic Hospital Cappagh.

Statistical analysis was performed using STATA^©^ Stata/IC 15.1 software, StataCorp, Texas. Descriptive statistics were performed for all demographic variables. The statistical test utilised was dependent on the variables being analysed. The chi-squared (*χ*^2^) test was used to compare categorical variables with more than 5 variables in each subgroup. The Fisher exact test was utilised when there were less than 5 variables per group. Two interval variables were analysed using simple regression analysis. Once the predictor variables were identified, all confounder variables were controlled for using a multivariate analysis. A *p*-value of < 0.05 was taken to be significant.

## Results

A total of 1351 cases were eligible for inclusion. There were 395 Stryker Exeter stems and 956 De Puy Charnley stems performed at this institution within the time period specified. There was a female preponderance of 55.8%. A Charnley stem was inserted in females in 536 cases, and an Exeter stem was inserted in 172 women. The mean BMI was 28.7 (Table 1). The majority of Charnley stems (82%) were inserted via the modified Hardinge approach whereas the majority of Exeter stems (62.4%) were inserted through the posterior approach (Table 2). Metal-on-polyethylene was the commonest bearing surface combination used for both stems. 10.2% of cases were inserted as hybrids.

**Table 1:**
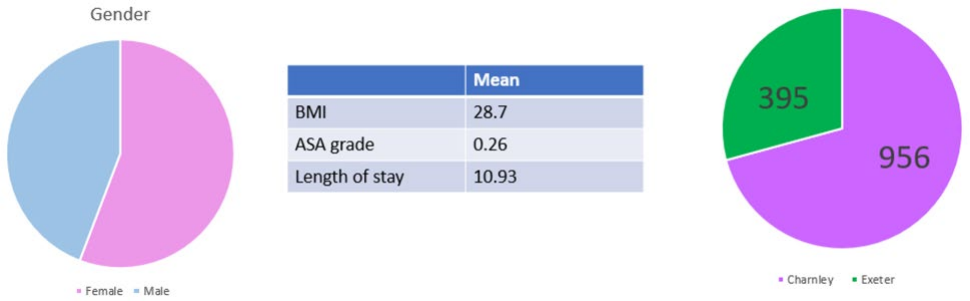
Demographics (according to Charnley vs Exeter)

**Table 2:**
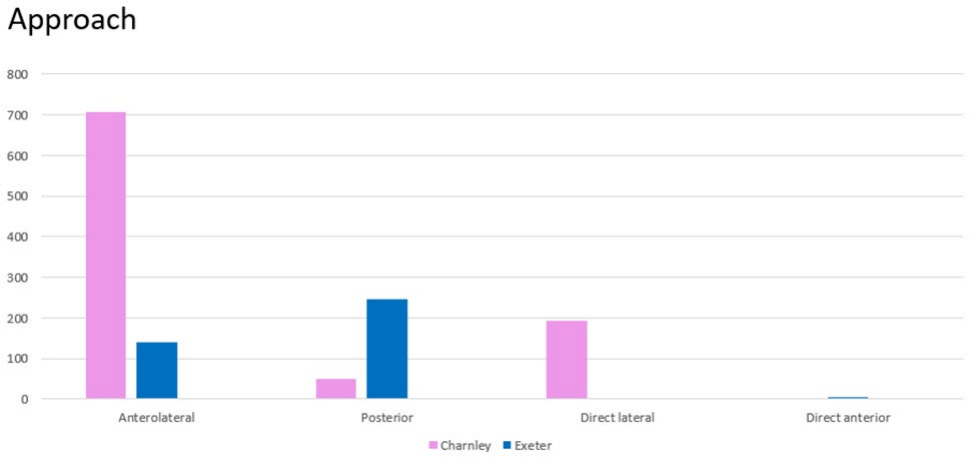
Approach

**Fig. 1:**
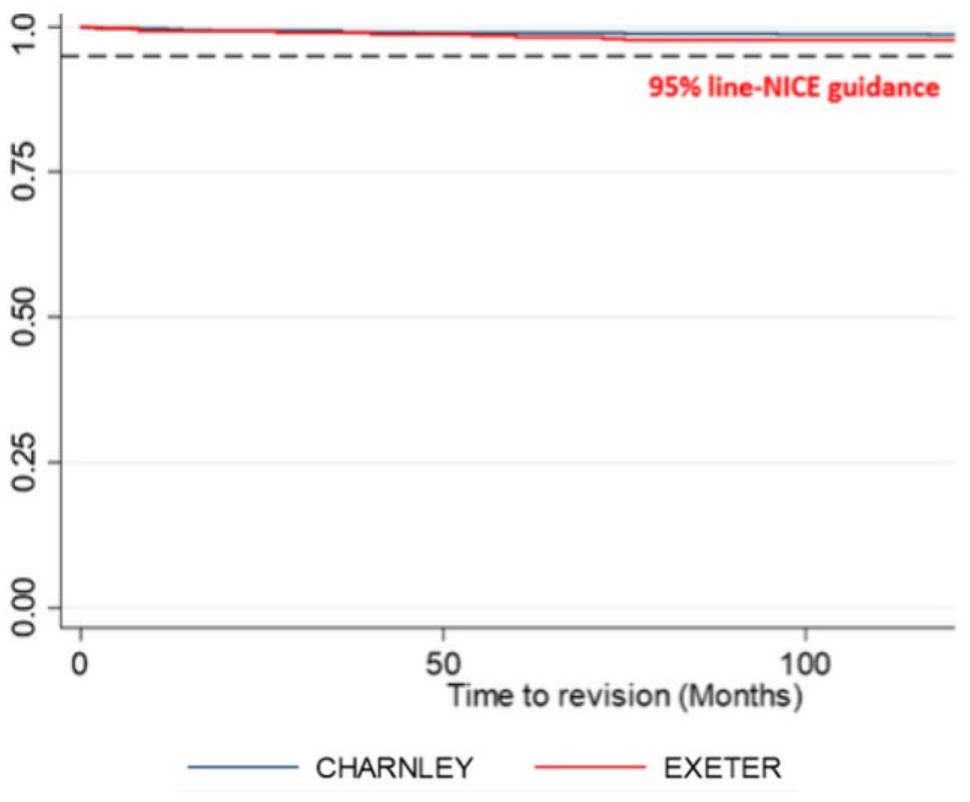
The Kaplan–Meier curve survivorship analysis

The overall all-cause stem revision rate at 10 years was 1.6% (*n* = 22). There was a 1.4% revision rate for all Charnley stems and a 2.3% revision rate for all Exeter stems with no significant difference noted between the two cohorts (*p* = 0.24) (Fig. 1). The overall mean time to revision was 38.3 months. The leading indication for revision was infection in 48% of cases; dislocation in 27% of cases; aseptic loosening (9%); and periprosthetic fracture (9%) were also found to be contributory (Table 3).

**Table 3:** Causes of revision

**Indication**	**Cases revised**	**Charnley**	**Exeter**
Infection	10 (46%)	6	4
Dislocation	6 (27%)	3	2
Loosening	2 (9%)	2	0
Peri prosthetic fracture	2 (9%)	0	1
Other	2 (9%)	2	2

*P* = 0.563

**Fig. 2:**
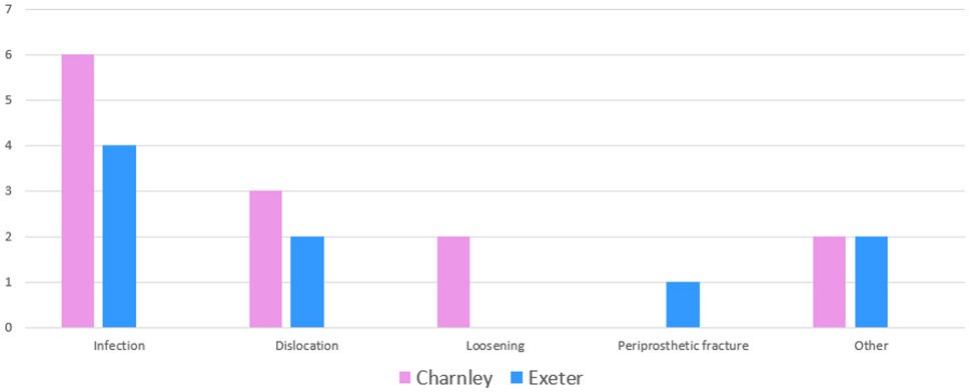
Cause of revision

Infection was found to be the indication for revision in the cases of 6 Charnley stems and 4 Exeter stems. There were 3 Charnley and 2 Exeter stems revised for instability. For aseptic loosening, there were 2 Charnley and no Exeter stems revised. One Exeter stem was revised for periprosthetic fracture with no Charnley stems revised for this reason (Fig. 2). WOMAC scores at 10 years were found to be higher for Charnley stems (mean 23.8, *σ* = 20.11) compared to Exeter stems (mean 19.78, *σ* = 20.72) (*p* = 0.016) (Table 4). The overall patient mortality rate was 7.54%. Mortality rates for patients with a Charnley stem were 7.6% compared to 7.34% in the Exeter stem at 10 years (Table 5). Loss to follow-up occurred in 41 cases (*N* = 29 Charnley, *N* = 12 Exeter).

**Table 4:** WOMAC scores

	Mean	Std Err	Std Dev
Charnley	23.81	1.133	20.11
Exeter	19.78	1.53	20.72

*P* < 0.1

**Table 5:** Mortality rates

	Charnley	Exeter	
Yes	73	29	102
No	883	366	1249
	956	395	1351

*P* > 0.05

## Discussion

Over the past decade, there has been a vogue towards the more widespread use of cementless stems with a coinciding decrease in the number of cemented implanted stems as reflected across international registry data [1, 14, 26]. There are numerous arguments supported by either side, such as higher rates of aseptic loosening in cemented THA [27] and higher rates of periprosthetic fractures with cementless stem use [28]. However, we postulate that survivorship of both designs of cemented stem consistently demonstrate successful outcomes and survivorship across 10-year follow-up. Although the Charnley stem is considered the more historic method of hip arthroplasty, we have demonstrated equal performance of the Charnley stem when we compared it to the use of the Exeter stem, with better performance across areas such as WOMAC score which did not quite reach statistical significance. The relevance of cemented implants moving forward is steadfast despite the surge in the popularity of cementless implants internationally. A meta-analysis in 2007 by Morshed et al. demonstrated no difference in survival between cemented and cementless prostheses [29]. Since then, many larger studies with longer follow-ups have been conducted and have produced varying results with a consensus yet to be reached on the optimal method of stem fixation [15–18].

Mid-term to long-term follow-up of the Charnley stem has demonstrated good functional results [30]. Caton et al. reported an 85% survivorship of the Charnley hip at 25 years [31]. Berry et al. reported similar 25-year survivorship rates at 86.5% [18]. They also demonstrated that the 25-year survivorship free of revision rate for aseptic loosening was poorer for each decade earlier in life at which the index procedure was performed; this ranged from 68.7% for patients who were less than 40 years of age to 100% for patients who were 80 years of age or older. This observation may explain why uncemented prostheses are now becoming more popular in younger patients worldwide [32]. Many surgeons have changed their preference with time [32]. Data from the several national arthroplasty registries show that cemented implants have a favourable outcome when revision of the implants is taken as the endpoint [9, 33, 34]. Malchau et al. examined the Swedish National Registry and found there to be a more favourable 10-year survival of cemented implants (94.8% vs 87.7%) [35]. These results were echoed by Danish arthroplasty registry findings, suggesting that cemented implants had similar lower revision rates [36].

With a 10-year all-cause revision rate of 1.6% for the 2 most popular cemented stems, our data supports the continued use of both of the studied designs of cemented stem in THA. Our data demonstrates an excellent performance of these 2 implants which compare extremely favourably to reported international revision rates.

Callaghan et al. found a 78% survivorship at 35 years for the Charnley THA [8]. Data of such longevity does not exist for uncemented fixation techniques yet. Supporting this, the Nordic Arthroplasty Registry demonstrated the survival of cemented implants for THR to be higher than that of uncemented implants. With a 93.8% 10-year survival rate for cemented implants in patients aged over 65, cemented stems were seen to be superior to uncemented stems with a survival of 92.9% [37]. Further subgroup analysis from this database showed the Charnley implant survival to be high (94.1% at 10 years) but slightly lower than that reported by the UK NJR (97% at 10 years) [38, 39]. The long-term survivorship of Exeter stems in our study was also excellent at 97.7%. This was noted to be comparable to rates reported by the NJR (10-year survival of 97.1%) [39]. This is also comparable to several studies examining for survivorship at 10 years; Westerman et al. reported survivorship of the stem, with revision for aseptic loosening as the endpoint, to be 100%. At 13.5 years, their survival rate for all-cause revision of the stem was reported as 96.8% [23].

The long-term performance of cemented THAs depends on many factors in addition to the implant, namely, the patient characteristics, the surgical approach, the cementing technique and the properties of the bone cement used [14]. The selection of bearing surfaces can contribute to rates of revision also [10, 40, 41]. In 2017, the New Zealand Registry demonstrated that ceramic-on-highly cross-linked polyethylene bearing surfaces provided the lowest all-cause revision rate [42]. In 2018, Sheridan et al. corroborated these results with data from our own regional arthroplasty registry. They demonstrated the lowest revision rates in ceramic-on-polyethylene bearings with a 0.9% all-cause revision for this bearing combination at 10 years [43].

The limitations of this study relate to the heterogenous nature of the group studied as it included the cases of multiple different surgeons within one centre, thereby encompassing different techniques and procedures. This does however add to the relatability of this study to practising arthroplasty units across the board. There are a number of confounding factors that we were unable to control for including the impact of bearing surfaces, individual surgical technique, approach and patient demographics.

## Conclusion

Cemented femoral stems have demonstrated excellent performance in our arthroplasty registry. Both the Charnley and Exeter stem provide similar outstanding overall survivorship at a 10-year follow-up. We suggest that the international increase in the use of uncemented THA may not be fully supported, and there remains a strong role for cemented implants in the future.
